# Effect of 1,5-anhydroglucitol levels on culprit plaque rupture in diabetic patients with acute coronary syndrome

**DOI:** 10.1186/s12933-020-01045-0

**Published:** 2020-05-30

**Authors:** Gong Su, Ming-Xi Gao, Gen-Ling Shi, Xi-Xi Dai, Wei-Feng Yao, Tao Zhang, Shao-Wei Zhuang

**Affiliations:** 1 Department of Cardiovascular Medicine, Shanghai General Hospital Baoshan Branch, No. 101 Tongtai North Road, Baoshan District, Shanghai, 200940 China; 2 Center of Cardiology, Beijing Anzhen Hospital, Capital Medical University, Beijing, 100029 China; 3 Department of Cardiovascular Medicine, The Seventh People’s Hospital, Shanghai University of Traditional Chinese Medicine, No. 358 Gaoqiaodatong Road, Pudong District, Shanghai, 200137 China

**Keywords:** 1,5-Anhydroglucitol, Plaque rupture, Acute coronary syndrome, Diabetes, Intravascular ultrasound

## Abstract

**Background:**

Postprandial hyperglycemia was reported to play a key role in established risk factors of coronary artery diseases (CAD) and cardiovascular events. Serum 1,5-anhydroglucitol (1,5-AG) levels are known to be a clinical marker of short-term postprandial glucose (PPG) excursions. Low serum 1,5-AG levels have been associated with occurrence of CAD. However, the relationship between 1,5-AG levels and coronary plaque rupture has not been fully elucidated. The aim of this study was to evaluate 1,5-AG as a predictor of coronary plaque rupture in diabetic patients with acute coronary syndrome (ACS).

**Methods:**

A total of 144 diabetic patients with ACS were included in this study. All patients underwent intravascular ultrasound examination, which revealed 49 patients with plaque rupture and 95 patients without plaque rupture in the culprit lesion. Fasting blood glucose (FBG), hemoglobin A_1c_ (HbA_1c_) and 1,5-AG levels were measured before coronary angiography. Fasting urinary 8-iso-prostaglandin F_2α_ (8-iso-PGF_2α_) level was measured and corrected by creatinine clearance.

**Results:**

Patients with ruptured plaque had significantly lower serum 1,5-AG levels, longer duration of diabetes, higher HbA_1c_ and FBG levels than patients without ruptured plaque in our study population. In multivariate analysis, low 1,5-AG levels were an independent predictor of plaque rupture (odds ratio 3.421; P = 0.005) in diabetic patients with ACS. The area under the receiver-operating characteristic curve for 1,5-AG (0.658, P = 0.002) to predict plaque rupture was superior to that for HbA_1c_ (0.587, P = 0.087). Levels of 1,5-AG were significantly correlated with urinary 8-iso-prostaglandin F_2α_ levels (r = − 0.234, P = 0.005).

**Conclusions:**

Serum 1,5-AG may identify high risk for coronary plaque rupture in diabetic patients with ACS, which suggests PPG excursions are related to the pathogenesis of plaque rupture in diabetes.

## Background

Acute coronary syndromes (ACS), including ST-elevation myocardial infarction (STEMI) and Non-ST-elevation acute coronary syndromes (NSTE-ACS), are a common cause of morbidity and mortality in individuals with diabetes. Autopsy data and intravascular imaging studies have showed that ACS results from spontaneous plaque rupture or erosion and subsequent thrombosis [1–3]. A meta-analysis, based on optical coherence tomography findings, showed the rate of plaque ruptures is 70.4% in STEMI patients, 55.6% in NSTEMI patients and 39.1% in unstable angina patients, respectively [4]. In an analysis of lesions from patients after sudden coronary death, ruptured plaque is recognized to be responsible for the most of cases of acute coronary thrombi [5]. Diabetic patients are at a high risk for cardiovascular events for having more vulnerable features in both culprit and non-culprit lesions compare to patients without diabetes [6]. Many researchers are attempting to find out what factors could affect coronary plaque rupture in diabetic patients for preventing critical outcomes.

Serum 1,5-anhydroglucitol (1,5-AG) level is a clinical marker to better reflect short-term postprandial hyperglycemia and glycemic variability (GV) than do hemoglobin A_1c_ (HbA_1c_) level [7]. Therefore, 1,5-AG levels may be associated with cardiovascular complications in diabetes. Indeed, some studies have reported that 1,5-AG levels are bound up with cardiovascular disease [8, 9]. Several clinical studies showed that 1,5-AG levels had utility to predict cardiovascular events in study population [10–12]. However, the association between 1,5-AG levels and coronary plaque rupture in diabetic patients with ACS is unclear. Intravascular ultrasound (IVUS) can provide detailed, high-quality tomographic images to detect plaque rupture in vivo [13]. In the present study, we investigated whether any relation exists between serum 1,5-AG level and ruptured plaque in culprit lesion identified by IVUS in diabetic patients with ACS.

## Methods

### Patient population and study design

This is a prospective observational study. We included 220 type 2 diabetes mellitus (T2DM) patients with ACS, who were admitted to Shanghai General Hospital Baoshan Branch and Beijing Anzhen Hospital between December 2018 and July 2019. All enrolled patients were admitted and underwent coronary angiography and IVUS in the culprit vessel. Patients with any of the following were excluded from the study: (1) totally occlusive lesions, (2) restenosis after stenting, (3) previous coronary artery bypass graft surgery, (4) severe heart failure (NYHA functional class III or above), renal failure (creatinine clearance < 30 mL/min), hepatic insufficiency, or infectious disease, (5) HbA_1c_ > 8%, (6) taking sodium-glucose cotransporter 2 inhibitor, and (7) insufficient clinical data. A total of 144 patients were included for analysis after excluding 76 patients who meeting the exclusion criteria. 49 patients had coronary plaque rupture in culprit lesion diagnosed by IVUS. ACS consisted of ST-segment elevation myocardial infarction (STEMI), non-ST-segment elevation myocardial infarction (NSTEMI) and unstable angina pectoris, which were defined according to 2013 ACCF/AHA guideline for the management of STEMI and 2014 ACC/AHA guideline for NSTE-ACS. T2DM was diagnosed according to the American Diabetes Association criteria or medical history and the use of insulin or glucose-lowering medication. Hypertension was defined as systolic blood pressure ≥ 140 mmHg and/or diastolic blood pressure ≥ 90 mmHg or treatment with oral antihypertensive drugs. Hyperlipidemia was diagnosed according to the modified National Cholesterol Education Program-Adult Treatment Panel III. The study protocol was approved beforehand by the Medical Ethics Committee of Shanghai General Hospital Baoshan Branch and the Medical Ethics Committee of Beijing Anzhen Hospital, and the procedures followed were in accordance with the institutional guidelines. The study complied with the Declaration of Helsinki, and informed consent was obtained from all patients.

### IVUS imaging protocol and analysis

All patients were performed with coronary angiography by standard Judkins technique. IVUS examination was performed using an IVUS system (iLAB™ Ultrasound Imaging System, Boston Scientific, USA) and a 40 MHz intravascular catheter (OptiCross™, Boston Scientific, USA) before any intervention. The IVUS catheter was advanced into the culprit vessel more than 10 mm beyond the culprit lesion and withdrawn at a pullback speed of 0.5 mm/s automatically. In this study, a culprit lesion was defined as the lesion related to the clinical event, as identified by both coronary angiography and electrocardiogram findings. A ruptured plaque was defined as the plaque contained a cavity that communicated with the lumen with an overlying residual fibrous cap fragment. A fragmented and loosely adherent plaque without a distinct cavity and without a fibrous cap fragment was not considered as a plaque rupture [14]. IVUS quantitative analysis was performed by two independent experienced interventional cardiologists who were blinded to the patients’ clinical information according to the criteria of the American College of Cardiology Clinical Expert Consensus Document on IVUS.

### Laboratory measurement

We collected blood samples and urine samples from patients after overnight fasting. Samples were stored at − 80 °C prior to analysis. Serum levels of 1,5-AG were measured by a colorimetric method (Nippon Kayaku, Tokyo, Japan) using a Lana 1,5-AG auto liquid automatic analyzer (JCA-BM 8060, JEOL Ltd., Tokyo, Japan). Serum concentration of hemoglobin A_1c_ (HbA_1c_) was determined by high-performance liquid chromatographic method (Tosoh HLC-723G7; Tosoh Corporation, Tokyo, Japan). The urinary 8-iso-prostaglandin F_2α_ (8-iso-PGF_2α_) levels were measured by a competitive enzyme-linked immunosorbent assay (Cayman Chemical, Ann Arbor, MI, USA) and corrected by creatinine clearance. The plasma concentration of fasting blood glucose (FBG), creatinine, total cholesterol (TC), high-density lipoprotein cholesterol (HDL-c), low-density lipoprotein cholesterol (LDL-c), triglyceride (TG), the high-sensitivity C-reactive protein (hs-CRP), N-terminal pro-brain natriuretic peptide (NT-proBNP), and troponin I (TnI) were measured. The non-HDL-c level was calculated as the TC level minus the HDL-c level.

### Statistical analysis

All statistical analyses were performed by using SPSS for Windows 24.0 (SPSS Inc, Chicago, IL, USA). All data were tested for normal distribution with the Kolmogorov–Smirnov test. Data are presented as mean with standard deviation (SD) for continuous distributed variables, frequencies and percentages for categorical variables, and median with 25% and 75% percentiles for abnormal distributed parameters. Differences between two groups were assessed by using the t-tests, Chi square, and Mann–Whitney rank analysis. Correlation between continuous variables was determined by Pearson correlation coefficients. Univariate and multivariate logistic regression analyses were performed in tow models to identify independent predictors for ruptured culprit plaque in study population. 1,5-AG level was included as a continuous variable in Model 1 and as a categorized variable (categorized into tertiles) in Model 2. The predictive value of 1,5-AG and HbA_1c_ for the presence of ruptured plaque in culprit lesion was calculated by constructing receiver-operating characteristic (ROC) curves. A value of P < 0.05 was considered statistically significant.

## Results

### Clinical characteristics of patients

**Fig. 1:**
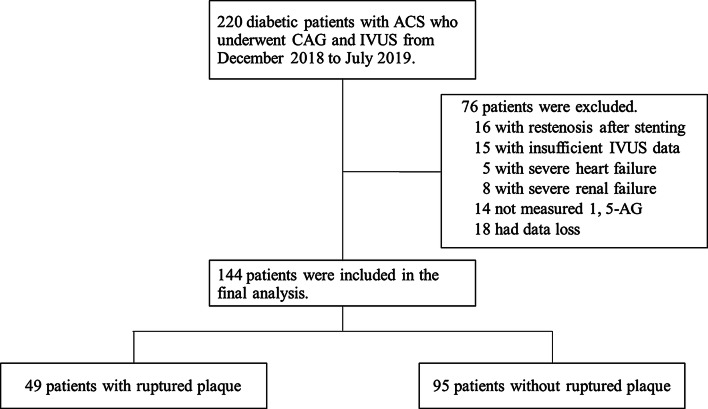
Flow chart of study population

During the study period, 220 diabetic patients with ACS underwent CAG and IVUS. We excluded 16 patients with restenosis after stenting, 15 patients with insufficient IVUS data, 5 patients with severe heart failure, 8 patients with renal failure, 14 patients without 1,5-AG data, and 18 patients with other data loss. Finally, a total of 144 patients were enrolled into the present study. Among of all subjects, 49 patients had culprit plaque rupture detected by IVUS (Rupture group), 95 patients had not (Non-rupture group) (Fig. 1). Compared to patients of non-rupture group, those patients with ruptured plaque had significantly lower 1,5-AG levels (10.5 ± 5.5 vs. 14.1 ± 7.7 μg/mL, P = 0.005), longer duration of diabetes [median (interquartile range): 4.2 (2.0, 5.2) vs. 2.5 (1.2, 4.8) years, P = 0.009], higher FBG (8.1 ± 3.0 vs. 7.1 ± 2.0 mmol/L, P = 0.023), HbA_1c_ (7.2 ± 0.5 vs. 7.0 ± 0.6%, P = 0.025), and non-HDL-c [4.39 (3.63, 4.99) vs. 3.83 (3.43, 4.36) mmol/L, P = 0.006] levels. Patients with plaque rupture also had higher hs-CRP [2.46 (1.61, 5.23) vs. 1.22 (0.84, 4.01), P = 0.014] and urinary 8-iso-PGF_2α_ (141.9 ± 67.2 vs. 116.1 ± 71.6 pmol/mmolCr, P = 0.038) levels than patients without plaque rupture. No significant differences were observed between two groups in terms of age, gender, hypertension, hyperlipidemia, body mass index (BMI), blood pressure, eGFR, and medicine treatments (Table 1).

**Table 1:** Clinical characteristics in the study population

Variables	Rupture	Non-rupture	P value
n	49	95	
Age (years)	59 ± 9	60 ± 10	0.474
Males	28 (57.1)	62 (65.3)	0.340
Current smoking	34 (69.4)	51 (53.7)	0.069
Hypertension	36 (73.5)	65 (68.4)	0.531
Hyperlipidemia	32 (65.3)	58 (61.1)	0.617
Duration of diabetes (years)	4.2 (2.0, 5.2)	2.5 (1.2, 4.8)	0.009
BMI (kg/m^2^)	26.4 ± 3.6	25.6 ± 3.5	0.110
LVEF (%)	59.5 ± 8.3	62.2 ± 9.2	0.092
eGFR (mL/min/1.73 m^2^)	84.6 ± 28.6	88.0 ± 30.8	0.491
SBP (mmHg)	130 ± 13	131 ± 16	0.612
DBP (mmHg)	76 ± 8	77 ± 10	0.638
TG (mmol/L)	1.93 (1.12, 2.13)	1.72 (0.94, 1.88)	0.079
HDL-C (mmol/L)	0.96 (0.82, 1.24)	1.02 (0.91, 1.23)	0.314
Non-HDL-C (mmol/L)	4.39 (3.63, 4.99)	3.83 (3.43, 4.36)	0.006
WBC (10^9^/L)	7.3 ± 1.6	7.2 ± 1.7	0.958
hs-CRP (mg/dL)	2.46 (1.61, 5.23)	1.22 (0.84, 4.01)	0.014
NT-proBNP	390 (155, 960)	362 (105, 890)	0.413
TnI	1.20 (0.11, 5.12)	0.91 (0.03, 2.62)	0.228
Urinary 8-iso-PGF_2α_ (pmol/mmolCr)	141.9 ± 67.2	116.1 ± 71.6	0.038
FBG (mmol/L)	8.1 ± 3.0	7.1 ± 2.0	0.023
HbA_1c_ (%)	7.2 ± 0.5	7.0 ± 0.6	0.025
1,5-AG (µg/mL)	10.5 ± 5.5	14.1 ± 7.7	0.005
Medications on admission			
Aspirin	36 (73.5)	71 (74.7)	0.869
Statins	27 (55.1)	62 (65.3)	0.234
ACEI/ARB	25 (51.0)	52 (54.7)	0.672
Oral antidiabetic drugs	31 (63.3)	64 (67.4)	0.622
Insulin	16 (32.7)	32 (33.7)	0.901

Data are given as number (percentage) for categorical variables and mean ± standard deviation or median (IQR) for continuous variables

*BMI* body mass index, *LVEF* left ventricular ejection fraction, *eGFR* estimated glomerular filtration rate, *SBP* systolic blood pressure, *DBP* diastolic blood pressure, *TG* triglycerides, *HDL*-*C* high-density lipoprotein cholesterol, *non*-*HDL*-*C* non-high-density lipoprotein cholesterol, *WBC* white blood cell, *hs*-*CRP* high-sensitivity C-reactive protein, *NT*-*proBNP* N-terminal pro-brain natriuretic peptide, *TnI* troponin I, *8*-*iso*-*PGF*_*2α*_ 8-iso-prostaglandin F_2α_, *FBG* fasting blood glucose, *HbA*_*1c*_ hemoglobin A_1c_, *1,5*-*AG* 1,5-anhydroglucitol, *ACEI/ARB* angiotensin-converting-enzyme inhibitor/angiotensin II receptor blocker

### Angiographic and IVUS results

Angiographic findings and IVUS analysis were summarized in Table 2. Culprit lesions of all 144 enrolled patients were evaluated by IVUS. Plaque rupture was observed in 49 patients (34%). There were no significant differences in culprit lesion location and three-vessel disease between plaque rupture and non-rupture groups. IVUS data showed there were not significant differences in lesion volume, length, plaque burden, external elastic membrane cross-sectional areas, lumen cross-sectional areas, plaque plus media cross-sectional areas, and remodeling index between patients with and without plaque rupture.

**Table 2:** Culprit lesion characteristics assessed by angiography and intravascular ultrasound

Variables	Rupture	Non-rupture	P value
n	49	95	
Angiographic analysis			
Culprit lesion			0.694
LM	2 (2.6)	5 (5.3)	
LAD	20 (34.2)	38 (40.4)	
LCX	9 (15.8)	16 (16.0)	
RCA	18 (47.4)	36 (38.3)	
Lesion location			0.579
Ostial	2(2.6)	6 (6.4)	
Proximal	18 (34.2)	33 (35.1)	
Mild	23 (52.6)	44 (46.8)	
Distal	6 (10.5)	12 (11.7)	
3-vessel disease	21 (42.9)	33 (34.7)	0.340
IVUS analysis			
EEM CSA (mm^2^)	20.9 ± 6.4	19.4 ± 5.4	0.106
Lumen CSA (mm^2^)	4.3 ± 1.4	4.2 ± 1.0	0.585
P&M CSA (mm^2^)	16.6 ± 5.9	15.1 ± 5.4	0.164
Plaque burden (%)	78.6 ± 8.0	76.8 ± 8.3	0.209
Length (mm)	19.0 ± 5.4	18.6 ± 6.8	0.688
Volume (mm^3^)	135.7 ± 55.3	127.4 ± 58.5	0.402
Remodeling index	1.01 ± 0.15	0.97 ± 0.17	0.196

Data are given as number (percentage) for categorical variables and mean ± standard deviation

*LM* left main coronary artery, *LAD* left anterior descending coronary artery, *LCX* left circumflex coronary artery, *RCA* right coronary artery, *IVUS* intravascular ultrasound, *EEM* external elastic membrane, *CSA* cross-sectional areas, *P&M* plaque plus media

### Relationship between 1,5-AG level and plaque rupture

**Fig. 2:**
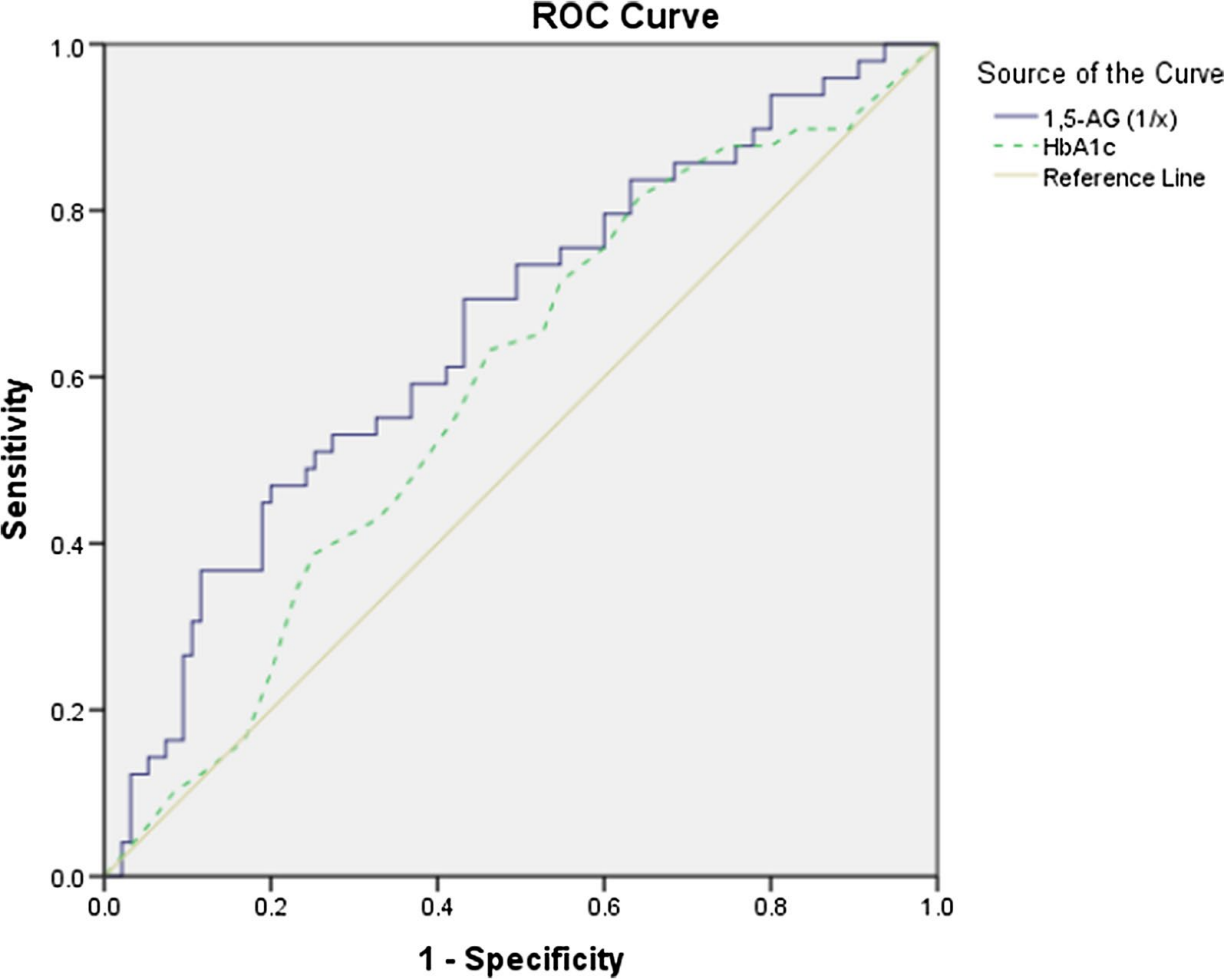
Receiver-operating characteristic curves of 1,5-AG and HbA_1c_ levels to predict coronary plaque rupture in diabetic patients with ACS. The areas under the curve of 1,5-AG (1/x) and HbA_1c_ levels were 0.658 (0.563–0.752, P = 0.002) and 0.587 (0.490–0.684, P = 0.087), respectively. *1,5*-*AG* 1,5-anhydro-d-glucitol, *HbA*_*1c*_ hemoglobin A_1c_

We performed univariate and multivariate analysis to determine independent predictors for plaque rupture in culprit lesion. For multivariable regression analysis in model 1, variables (age, gender, current smoking, duration of diabetes, body mass index (BMI), left ventricular ejection fraction (LVEF), TG, non-HDL-C, hs-CRP, urinary 8-iso-PGF_2α_, 1,5-AG, FBG and HbA_1c_] were entered into the univariate regression analysis, and variables with P < 0.10 (current smoking, duration of diabetes, LVEF, TG, non-HDL-C, urinary 8-iso-PGF_2α_, 1,5-AG, FBG and HbA_1c_) and forced inclusion variables that were considered as important predictors of plaque rupture or associated with 1,5-AG (age, gender, BMI) were further entered into the multivariable regression model. The result showed that 1,5-AG (OR 0.916, 95% CI 0.852–0.985, P = 0.018) as well as other variables (current smoking and non-HDL-C) were associated with risk of culprit plaque rupture (Table 3). In model 2, age (≥ 65 years), duration of diabetes (upper tertile, ≥ 4.8 years), BMI (> 30 kg/m^2^), LVEF (< 40%), TG (> 1.70 mmol/L), non-HDL-C (≥ 4.1 mmol/L), hs-CRP (> 3 mg/L), 1,5-AG (lower tertile, < 9.78 µg/mL), FBG (≥ 7.0 mmol/L), HbA_1c_ (> 7%) and urinary 8-iso-PGF_2α_ (upper tertile, > 140 pmol/mmolCr) were included as categorized variables. Low level of 1,5-AG (OR 3.421, 95% CI 1.446–8.092, P = 0.005), current smoking (OR 3.529, 95% CI 1.292–9.638, P = 0.014), high level of non-HDL-c (OR 4.857, 95% CI 1.871–12.61, P = 0.001) and HbA_1c_ (OR 2.458, 95% CI 1.081–5.586, P = 0.032) were significantly associated with culprit plaque rupture in diabetic patients with ACS (Table 3). We constructed a ROC curve for predicting ruptured culprit plaque by 1,5-AG and HbA_1c_ levels in patients. The area under the ROC curve for reciprocal of 1,5-AG (0.658, 95% CI 0.563–0.752, P = 0.002) was significantly superior to that for HbA_1c_ (0.587, 95% CI 0.490–0.684, P = 0.087) (Fig. 2). The optimal cut-off value for 1,5-AG to predict culprit plaque rupture was 11.8 µg/mL (69.4% sensitivity and 59.8% specificity).

**Table 3:** Independent predictors for ruptured culprit plaque

Model 1	Univariate		Multivariate		Model 2	Univariate		Multivariate	
	OR (95% CI)	P value	OR (95% CI)	P value		OR (95% CI)	P value	OR (95% CI)	P value
Age	0.992 (0.958, 1.028)	0.672			Age (≥ 65 years)	0.869 (0.414, 1.824)	0.710		
Female	1.305 (0.608, 2.801)	0.494			Female	1.305 (0.608, 2.801)	0.494		
Current smoking	2.278 (0.961, 5.398)	0.062	2.647 (1.018, 6.883)	0.046	Current smoking	2.278 (0.961, 5.398)	0.062	3.529 (1.292, 9.638)	0.014
Duration of diabetes	1.059 (0.991, 1.130)	0.090			Duration of diabetes (Upper tertile, ≥ 4.8 years)	1.773 (0.851, 3.695)	0.126		
Body mass index	1.102 (0.978, 1.243)	0.111			Body mass index (> 30 kg/m^2^)	1.956 (0.943, 4.055)	0.071		
LVEF	0.968 (0.932, 1.006)	0.095			LVEF (< 40%)	1.718 (0.659, 4.479)	0.268		
TG	1.353 (0.961, 1.906)	0.084			TG (> 1.70 mmol/L)	1.681 (0.810, 3.490)	0.164		
Non-HDL-C	1.904 (1.233, 2.940)	0.004	1.983 (1.236, 3.183)	0.005	non-HDL-C (≥ 4.1 mmol/L)	4.016 (1.736, 9.293)	0.001	4.857 (1.871, 12.61)	0.001
hs-CRP	1.007 (0.940, 1.080)	0.837			hs-CRP (> 3 mg/L)	1.372 (0.668, 2.817)	0.389		
1,5-AG	0.914 (0.856, 0.976)	0.007	0.916 (0.852, 0.985)	0.018	1, 5-AG (Lower tertile, < 9.78 µg/mL)	3.631 (1.752, 7.524)	0.001	3.421 (1.446, 8.092)	0.005
FBG	1.176 (1.019, 1.357)	0.026			FBG (≥ 7.0 mmol/L)	1.367 (0.685, 2.729)	0.375		
HbA_1c_	1.989 (1.083, 3.653)	0.027			HbA_1c_ (> 7%)	1.996 (0.984, 4.048)	0.055	2.458 (1.081, 5.586)	0.032
Urinary 8-iso-PGF_2α_	1.005 (1.000, 1.010)	0.042			Urinary 8-iso-PGF_2α_ (Upper tertile, > 140 pmol/mmolCr)	2.493 (1.222, 5.085)	0.012		

Model 1: Age, duration of diabetes, body mass index, LVEF, TG, non-HDL-C, hs-CRP, 1,5-AG, FBG, HbA_1c_ and urinary 8-iso-PGF_2α_ were included as continuous variables. Model 2: All variables were included as categorized variables

*LVEF* left ventricular ejection fraction, *TG* triglycerides, *non*-*HDL*-*C* non-high-density lipoprotein cholesterol, *hs*-*CRP* high-sensitivity C-reactive protein, *1,5*-*AG* 1,5-anhydroglucitol, *FBG* fasting blood glucose, *HbA*_*1c*_ hemoglobin A_1c_, *8*-*iso*-*PGF*_*2α*_ 8-iso-prostaglandin F_2α_

### Correlation between serum 1,5-AG level and urinary 8-iso-PGF_2α_, or other ACS biomarkers

**Fig. 3:**
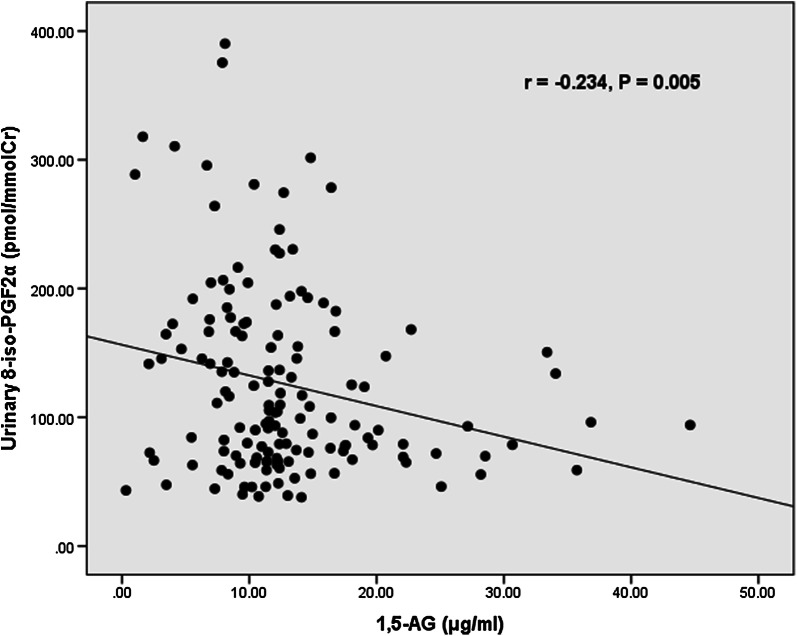
Correlations between serum 1,5-AG level and urinary 8-iso-PGF_2α_ level in diabetic patients with acute coronary syndrome. *1,5*-*AG* 1,5-anhydro-d-glucitol, *8*-*iso*-*PGF*_*2α*_ 8-iso-prostaglandin F_2α_

A significant negative correlation was noted between serum level of 1,5-AG and urinary 8-iso-PGF_2α_ level (r = − 0.234, P = 0.005) (Fig. 3). There was no significant correlation between the level of 1,5-AG and hs-CRP (r = − 0.116, P = 0.165), TnI (r = − 0.012, P = 0.887), or NT-proBNP (r = − 0.011, P = 0.898). The correlation between HbA_1c_ level and urinary 8-iso-PGF_2α_ level was also not significant (r = 0.076, P = 0.368).

## Discussion

Most cases of sudden cardiac death and myocardial infarction arise from thrombotic coronary occlusion following coronary plaque rupture. Diabetic patients had more plaque ruptures and thrombus than non-diabetic patients in ACS, which may be associated with the greater rates of cardiovascular events in diabetes [15]. However, available screening and diagnostic methods are insufficient to identify the victims before the event occurs. The search for noninvasive approach to detect the plaque rupture was encouraged to perform. In our present study, the principal result shows that serum 1,5-AG, as a biomarker of short-term postprandial hyperglycemia and GV, might be an important surrogate marker of coronary plaque rupture in diabetic patients with ACS.

### 1,5-AG levels and coronary plaque rupture

1,5-AG is a naturally occurring 1-deoxy form of glucose. As glucose levels surpass the renal threshold for glucosuria (generally around 10 mmol/L), 1,5-AG is excreted in the urine leading to a rapid reduction in serum levels [16]. Therefore, poor glycemic control is associated with low, rather than high, serum 1,5-AG levels. Unlike HbA_1c_, 1,5-AG values are not affected by mean erythrocyte age and can accurately reflect the glycemic control in hemolytic patients [17]. On the other hand, 1,5-AG levels can also reflect the status of gestational DM in pregnant women and may anticipate the development of gestational DM [18]. In the present study, we used IVUS to identify plaque rupture in culprit lesion of diabetic patients with ACS and found that the 1,5-AG levels were significantly lower in patients with ruptured plaque than in patients without ruptured plaque. Meanwhile, patients with ruptured plaque had higher HbA_1c_, FBG, and non-HDL-c levels compared to patients with non-ruptured plaque. This result indicates that diabetic patients with ruptured culprit plaque had worse glycometabolic and lipidemic disorders. In accordance with some recent in vivo studies [19, 20], no differences were observed for age and sex between the patients with or without culprit plaque rupture. In model 1 and model 2, univariate and multivariate logistic regression analyses showed that low 1,5-AG, high non-HDL-c levels and current smoking were independent predictors of plaque rupture of culprit lesion in diabetic patients with ACS. These results indicate that poor glycemic control and dyslipidemia may be associated with the coronary plaque rupture in diabetes. Sheng et al. reported that increased duration of DM combined with higher HbA_1c_ levels influences culprit-plaque characteristics in patients with DM who suffer AMI [21]. In the present study, patients with ruptured plaque had longer duration of DM and higher HbA_1c_ levels than patients without ruptured plaque. The level of HbA_1c_ > 7% is associated with an increased risk of culprit plaque rupture. Moreover, the ROC curve analysis showed 1,5-AG displayed more significant value in predicting plaque rupture than HbA_1c_. Preliminary data have shown that 1,5-AG could be expected to best reflect postprandial hyperglycemia in moderately controlled patients and was more sensitive and specific than HbA_1c_ [22]. Furthermore, as PPG increments are the major contributors to GV in T2DM, 1,5-AG may be particularly suited for monitoring postprandial hyperglycemic excursions [23]. Unlike HbA_1c_, 1,5-AG is not affected by hypoglycemia. As a result, 1,5-AG appears to differentiate patients with extensive PPG excursions despite having similar HbA_1c_ levels. Selvin et al. reported that patients with low 1,5-AG levels had an increased risk of coronary artery disease, stroke, heart failure, and death compared to patients with high 1,5-AG levels [10]. Takahashi et al. reported that low and exacerbated levels of 1,5-AG are associated with cardiac mortality in ACS patients [11]. The study of Fujiwara et al. showed that 1,5-AG was associated with the presence of de novo coronary artery disease in both well-controlled diabetic and non-diabetic patients [8]. Low 1,5-AG level had been found to be associated with coronary artery calcification, which may be related to the coronary plaque vulnerability [24, 25]. The current study is the first to report that 1,5-AG levels are significantly associated with coronary plaque rupture in diabetic patients with ACS. These findings may partly explain the results of previous studies that 1,5-AG levels were associated with cardiovascular outcomes and support the hypothesis that PPG excursions is strongly associated with the atherosclerotic vulnerable plaque process.

### Mechanism for PPG excursions affecting plaque rupture

Although the identified role of PPG excursions in the pathogenesis of plaque rupture has not been clarified, oxidative stress, inflammation and endothelial dysfunction may be involved in the process. It was demonstrated that glucose excursions increased oxidative stress than chronic hyperglycemia in T2DM. Ceriello et al. showed that targeting postprandial hyperglycemia has the potential to reduce oxidative stress [26]. We have recently reported that glycemic variability, a component of which is PPG excursions, was significantly correlated with oxidative stress measured as urinary 8-iso-PGF_2α_ in patients with ACS [27]. Urinary 8-iso-PGF_2α_ have been proved to be the most reliable marker to assess lipid peroxidation, which is a key mechanism for the development of atherosclerotic plaques in humans. The present study showed that serum 1,5-AG level, but not HbA_1c_, was strongly correlated with urinary 8-iso-PGF_2α_ level in diabetic patients with ACS. This is in accordance with the previous report of Kohata et al. that 1,5-AG is the strong correlate of oxidative stress in patients with T2DM [28], and it suggests that PPG excursions can be more important than mean glucose to induce oxidative stress in diabetes. It has been demonstrated that oxidative stress plays a key role in atherosclerotic plaque progression [29]. Our previous study showed that increased urinary 8-iso-PGF_2α_ levels were closely associated with greater absolute and percent necrotic core volumes of coronary lesions in diabetic patients [30]. In a pathological study by Nishibe et al., 8-iso-PGF_2α_ was found enriched in coronary plaque specimens especially from vulnerable patients, suggesting a crucial role of free radicals in the formation of vulnerable plaques [31]. Yura et al. reported that 8-iso-PGF_2α_ per se could stimulate endothelin-1 mRNA and protein expression in bovine aortic endothelial cells [32]. Endothelin-1 may cause the stimulation of vascular smooth muscle proliferation and formation of macrophage-rich atherosclerotic plaques. In the study of Esposito et al., the results suggested that acute hyperglycemia, and not sustained elevation of blood glucose levels, could exaggerate inflammation by an oxidative mechanism [33]. Teraguchi et al. reported that dynamic glucose fluctuation was positively and significantly associated with CD14^bright^ CD16+ monocytes levels and might be related to coronary plaque rupture in patients with acute myocardial infarction [34]. All these findings suggest that postprandial hyperglycemic excursions may be involved in progression and destabilization of coronary plaques through the preferential increase in oxidative stress, proinflammatory cytokines, and endothelial dysfunction. Optimizing PPG excursions management may be helpful to prevent the rupture of coronary plaque in diabetic patients.

### Study limitations

Several study limitations should be considered in the interpretation of the results. First, the sample size was relatively small, so that it may have influenced the results and the statistical analyses. Second, because we evaluated only limited patients who underwent IVUS and didn’t meet any exclusion criteria, our results could have been affected by selection bias and cannot be generalized to all patients. Third, the assessment of plaque rupture was made by IVUS in this study. Although it has been demonstrated that IVUS can provide detailed, high-quality tomographic images to detect plaque rupture, it might be likely that some plaque ruptures were undetected. More detailed plaque morphology could be obtained by combining high definition IVUS with optical coherence tomography (OCT), virtually increasing accuracy for plaque rupture detection. Finally, this is an observational study. The observational nature of analysis means that we cannot infer causality in the associations we have demonstrated. Future longitudinal and prospective studies are needed to address these issues.

## Conclusions

Serum 1,5-AG displayed significant value in predicting culprit plaque rupture in diabetic patients with ACS. This suggests that PPG excursions are related to the pathogenesis of plaque rupture in diabetes. The manipulation of PPG excursions may provide a potential therapeutic target for preventing plaque rupture.

## Data Availability

The datasets used and/or analyzed during the current study are available from the corresponding author on reasonable request.

## Abbreviations

1,5-AG: 1,5-Anhydroglucitol
PPG: Postprandial glucose
GV: Glycemic variability
ACS: Acute coronary syndrome
T2DM: Type 2 diabetes mellitus
IVUS: Intravascular ultrasound
8-iso-PGF_2α_: 8-Iso-prostaglandin F_2α_
FBG: Fasting blood glucose
HbA_1c_: Hemoglobin A_1c_
BMI: Body mass index
non-HDL-C: Non-high-density lipoprotein cholesterol
hs-CRP: High-sensitivity C-reactive protein
NT-proBNP: N-terminal pro-brain natriuretic peptide
TnI: Troponin I
LVEF: Left ventricular ejection fraction
